# Enhancing Agrobacterium-Mediated Hairy-Root Transformation Efficiency in Peanut Through the Application of *GRF*, *GIF* and *WOX* Genes

**DOI:** 10.3390/plants15121889

**Published:** 2026-06-18

**Authors:** Qianqian Zhang, Yuanyuan Cui, Fangjun Chen, Xiaoqin Liu

**Affiliations:** Peking University Institute of Advanced Agricultural Sciences, Shandong Laboratory for Advanced Agricultural Sciences at Weifang, Weifang 261325, China; qianqian.zhang@pku-iaas.edu.cn (Q.Z.); yuanyuan-cui@pku-iaas.edu.cn (Y.C.); fangjun.chen@pku-iaas.edu.cn (F.C.)

**Keywords:** hairy roots, *GRF*, *GIF*, *WOX*

## Abstract

Peanut (*Arachis hypogaea* L.) is a major oil and economic crop, yet genetic transformation remains inefficient and time-consuming, hindering functional genomics and molecular breeding. In this study, we found that the use of *GRF*, *GIF* and *WOX* genes improved the efficiency of Agrobacterium-mediated peanut hairy-root transformation. Here, we identified multiple peanut Growth-Regulating Factor (*GRF*) genes, GRF-Interacting Factor (*GRF-GIF*) fusion genes and WUSCHEL-related homeobox (*WOX*) genes, constructed high-expression vectors, and delivered them into *A. rhizogenes* to infect 3–5 cm peanut stem segments cut from 30-day-old seedlings. Statistical analysis of the data showed that, relative to the empty-vector control, expression of these developmental regulators markedly enhanced hairy-root growth: the number of roots per explant increased by 1.3–2.4-fold. Observations using reporter constructs showed that growth factors (besides 2S-PL-GUS and GRF-2A-T-GUS) improved the transformation efficiency of hairy roots, among which the highest transformation efficiency of GRF-2A (396)-GIF-GUS was 85.14 ± 2.94%. Collectively, these findings provide an efficient and rapid platform for the study of peanut gene function.

## 1. Introduction

Peanut (*Arachis hypogaea* L.) is an important oil crop cultivated in more than 100 countries [1]. Its seeds are rich in lipids, proteins, folate, tocopherols, phytosterols, and polyphenols [2,3]. Nowadays, China ranks first in the world in terms of total peanut production, accounting for more than half of the country’s total oil crop production. They play an important role in ensuring the safety of edible oil in China [4]. With rapid advances in biotechnology, genetic engineering has become a powerful approach for crop improvement [5,6]. However, the peanut genetic transformation system still faces problems of low efficiency and poor stability, which seriously restricts the research on gene function and the process of transgenic breeding [7]. *Agrobacterium rhizogenes*-induced hairy roots are fast-growing roots generated after infection and have been widely used for gene-function studies, secondary metabolite production, and trait improvement [8]. This system has been widely applied in peanut. For example, Nanjareddy et al. established a rapid, simplified, and cost-effective composite plant system in peanut using Agrobacterium rhizogenes-mediated induction, which can be used for generating transgenic hairy roots, analyzing root branching, and studying arbuscular mycorrhizal fungal symbiosis [9]. In peanut, the system is still constrained by low induction efficiency, insufficient root biomass, and large variability among explants [2].

Growth-Regulating Factors (*GRFs*) are plant-specific transcription factors that promote growth and organ development. GRF-Interacting Factors (*GIFs)* act as transcriptional co-regulators and are essential for cell proliferation, organogenesis, and regeneration [10,11]. Overexpressing *AtGRF1* or *AtGRF2* in *Arabidopsis* increases leaf size, and overexpression of the pear gene *PbGRF18* in tomato can promote fruit development and sugar accumulation [12,13]. Notably, *GRF-GIF* fusion (chimera) constructs can enhance transformation and regeneration efficiency and shorten regeneration cycles in crops such as maize, wheat, and soybean [14,15,16]. In peanut, researchers identified 22 GRF genes and demonstrated that *AhGRFi* is involved in seedling root development in Arabidopsis by regulating the expression of auxin-responsive genes [17]. In peanut, however, the application of *GRF/GIF* has rarely been reported, highlighting the need to evaluate *GRF-GIF* tools and develop a more efficient and practical transformation platform.

WOX proteins are key transcription factors that maintain meristem activity and regulate organogenesis by controlling cell fate and re-differentiation [18]. Target of rapamycin (*TOR*) is a conserved central growth regulator in eukaryotes; research shows that *PvTOR* is a key player in regulating arbuscule development during Arbuscular mycorrhizal symbiosis in Phaseolus vulgaris [19]. The expression of *WOX* genes or its combination can improve the transgenic efficiency of plants, and its effect on plant development has great potential research value [20]. In *Arabidopsis*, the *WOX* family (*WUS* and *WOX1–WOX14*) participates in development of multiple organs, including roots, stems, leaves, flowers, and fruits [21]. *WOX11* directly activates *LBD16* to initiate root primordia and thereby promotes lateral and adventitious root formation [22]. In woody species, *BpWOX11* (Betula platyphylla) increases adventitious rooting of cuttings, and *JrWOX5* (walnut) promotes root formation and influences plant architecture [23,24]. Despite these advances, the use of *WOX* genes to enhance induction performance in peanut hairy-root systems remains limited, and a *WOX*-based tool that improves rooting efficiency would be valuable.

To test whether species-native peanut developmental regulators could improve *A. rhizogenes*-mediated hairy-root transformation in peanut, we selected endogenous peanut homologs because they may be more compatible with the peanut cellular and regulatory background than regulators from distantly related species. However, we recognize that overexpression of developmental regulators, whether heterologous or endogenous, may cause undesirable effects, including pleiotropic developmental phenotypes, abnormal organogenesis, impaired regeneration, and transgene-induced silencing or cosuppression of endogenous homologs [25]. To evaluate whether peanut-native developmental regulators can improve *A. rhizogenes*-mediated hairy-root transformation, we identified peanut *GRF*, *GIF*, and *WOX* homologs and generated single-gene and combinatorial expression constructs. Although overexpression of developmental regulators may cause undesirable effects, including pleiotropic development or transgene-induced silencing, several tested constructs enhanced hairy-root regeneration and transformation performance in a construct-dependent manner. These results provide useful candidate modules for improving peanut hairy-root transformation and gene-function studies.

## 2. Results

### 2.1. Phylogenetic Analysis and Selection of Peanut GRF/GIF/WOX Candidates and Construction of Expression Vectors

**Figure 1:**
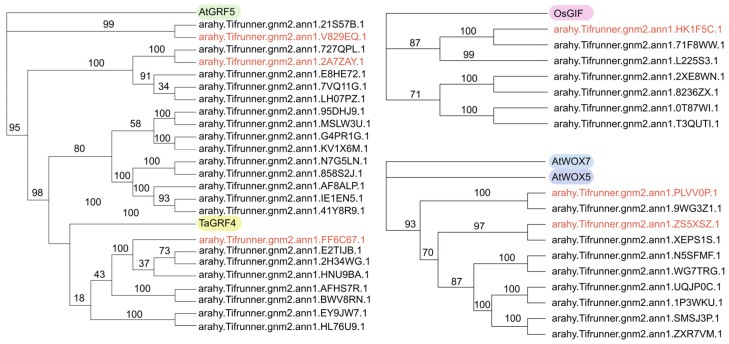
Phylogenetic analysis and selection of representative GRF, GIF and WOX proteins from Arachis hypogaea cv. Tifrunner and reference species (*Arabidopsis*, rice, and wheat). Protein sequences were aligned and used to infer the tree. Tifrunner genes are labeled with their genome annotation IDs (arahy.Tifrunner.gnm2.ann1.). Candidate regulators selected for vector construction and functional testing are highlighted, including *GRF-V829EQ*, *GRF-2A7ZAY* and *GRF-FF6C67*; *GIF-HK1F5C*; *WOX-PLVV0P* and *WOX-ZS5XSZ*), alongside known reference regulators (e.g., *AtGRF5/TaGRF4*, *OsGIF*, *AtWOX5*).

To identify peanut developmental regulators with potential utility in transformation enhancement, we performed phylogenetic analyses of GRF, GIF and WOX proteins from peanut and representative model/crop species. In the *GRF* clade, several peanut proteins grouped closely with established regulators such as *AtGRF5* and *TaGRF4*, supporting functional conservation and motivating the selection of *GRF-V829EQ*, *GRF-2A7ZAY* and *GRF-FF6C67* for downstream testing (Figure 1). Similarly, the peanut candidate *GIF-HK1F5C* clustered with the reference *OsGIF*, indicating it represents a likely functional *GIF* ortholog (Figure 1). For the *WOX* family, *WOX-PLVV0P* and an additional candidate, *WOX-ZS5XSZ* grouped with the reference *AtWOX5*, suggesting conserved roles associated with meristematic or regenerative competence (Figure 1).

**Figure 2:**
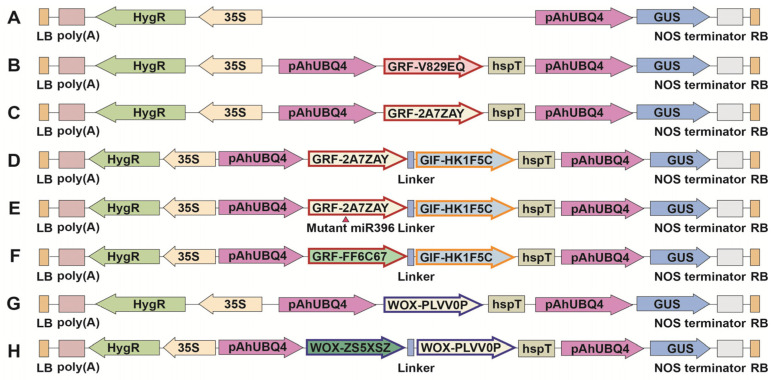
Schematic representation of binary T-DNA vectors used for peanut hairy-root transformation assays. (**A**): empty vector control; (**B**): *GRF-V829EQ* vector; (**C**): *GRF-2A7ZAY* vector; (**D**): *GRF-2A7ZAY-GIF-HK1F5C* vector; (**E**): *GRF-2A7ZAY_Mutant-GIF-HK1F5C* vector; (**F**): *GRF-FF6C67-GIF-HK1F5C* vector; (**G**): *WOX-PLVV0P* vector; (**H**): *WOX-ZS5XSZ-WOX-PLVV0P* vector. All constructs contain a *35S*-driven hygromycin resistance marker (Hyg) for selection and a *pAhUBQ4*-driven GUS reporter followed by a *NOS* terminator for scoring transformed roots. Developmental regulator (*DR*) expression cassettes were driven by *pAhUBQ4* and include single-gene constructs (e.g., *GRF-V829EQ*, *GRF-2A7ZAY*, *WOX-PLVV0P*), combinatorial modules for co-expression of GRF and GIF (linked by a 2A peptide, as indicated), a miR396-modified *GRF* module (denoted “Mutant miR396”) assembled with *GIF-HK1F5C*, and a dual-*WOX* construct (*WOX-ZS5XSZ-WOX-PLVV0P*) connected by a linker. LB, left border; RB, right border.

Based on these phylogenetic relationships and the tissue-specific expression profiles, we selected *GRF-V829EQ*, *GRF-2A7ZAY*, *GRF-FF6C67*, *GIF-HK1F5C*, *WOX-PLVV0P*, and *WOX-ZS5XSZ* for different construct combinations (Figure 1 and Figure S1). Based on the phylogenetic analysis, *GRF-V829EQ*, *GRF-2A7ZAY*, and *GRF-FF6C67* were selected because they clustered within the *GRF* clade containing known growth- and regeneration-related regulators, including *AtGRF5* and *TaGRF4*. *GIF-HK1F5C* was selected because it clustered with *OsGIF*, indicating that it is a likely functional GIF ortholog. *WOX-PLVV0P* and *WOX-ZS5XSZ* were selected because they grouped with *AtWOX5*, suggesting potential roles associated with meristematic activity and root developmental competence. The tissue-specific expression profiles in Figure S1 further supported the selection of the *GRF* and *GIF* candidates. *GIF-HK1F5C* was broadly and highly expressed in peanut reproductive and pod-related tissues, including aerial peg, subterranean peg, expanding pod, pod pericarp, seed, and seed-pericarp developmental stages, with expression values of 59.54 in the subterranean peg, 68.29 in expanding pod, and 56.90 in pod pericarp at stage 3. *GRF-FF6C67* and *GRF-2A7ZAY* showed preferential expression during seed development, with *GRF-FF6C67* reaching 20.17, 28.31, and 30.00 at SdPt7, SdPt8, and SdPt10, respectively, and *GRF-2A7ZAY* reaching 12.67, 27.73, and 22.88 at SdPt6, SdPt7, and SdPt8, respectively. *GRF-V829EQ* showed lower but detectable expression in seed-related tissues, with its highest expression at SdPt10. Together, the phylogenetic relationships and tissue-expression patterns supported the use of these genes for single-gene and combinatorial construct design. We generated a compact vector set to evaluate single and combinatorial regulator modules in peanut hairy-root assays. All constructs shared a common architecture including a *35S*::HygR selection cassette and a *pAhUBQ4*::GUS reporter cassette for rapid quantification of transformation output (Figure 2). Regulator expression cassettes were driven by *pAhUBQ4*, including single-gene constructs (*GRF-V829EQ*, *GRF-2A7ZAY*, *WOX-PLVV0P*) as well as combinatorial modules, notably GRF-GIF co-expression constructs (*GRF-2A7ZAY-GIF-HK1F5C* and *GRF-FF6C67-GIF-HK1F5C*) and a miR396-modified *GRF-GIF* module to mitigate miR396-associated repression (Figure 2). Together, these designs established a standardized genetic toolkit for comparing developmental regulator modules under the same promoter and reporter framework (Figure 2 and Table S1).

### 2.2. Induction of Hairy Roots by GRF, GIF and WOX Genes

To evaluate their potential effects in peanut hairy-root transformation, we quantified the number of regenerated hairy roots per explant at different time points after infection and the root transformation rate was calculated using the GUS histochemical staining test 2 weeks after the explants were transferred to MS medium.

As shown in Table 1, A (the empty-vector control) produced no visible regenerated roots during the first week, but gradually developed roots over time, reaching 18.67 ± 1.53 roots per explant after four weeks, with a transformation rate of 64.58 ± 5.44%. Different developmental regulator constructs showed distinct effects on both the number of regenerated roots per explant and the transformation rate. Among all constructs, E (*GRF-2A7ZAY_Mutant-GIF-HK1F5C* vector) showed the strongest overall performance. It produced the highest number of regenerated roots at each time point, increasing from 6.33 ± 1.53 roots per explant after one week to 44.67 ± 1.53 roots per explant after four weeks. Consistently, E also showed the highest transformation rate (85.14 ± 2.94%), which was significantly higher than that of the empty-vector control.

Constructs D (*GRF-2A7ZAY-GIF-HK1F5C* vector) and F (*GRF-FF6C67-GIF-HK1F5C* vector) also showed improved performance compared with the control. After four weeks, they produced 32.00 ± 1.00 and 31.33 ± 1.53 roots per explant, respectively, and their transformation rates reached 78.18 ± 2.45% and 76.72 ± 3.69%, respectively. Construct B (*GRF-V829EQ* vector) promoted the number of regenerated roots per explant, reaching 34.00 ± 1.00 roots per explant after four weeks, although its transformation rate (67.69 ± 2.00%) was only slightly higher than that of the empty-vector control. In contrast, constructs C (*GRF-2A7ZAY* vector), G (*WOX-PLVV0P* vector), and H (*WOX-ZS5XSZ-WOX-PLVV0P* vector) showed relatively weaker effects, with four-week root numbers of 27.00 ± 1.00, 27.00 ± 1.00, and 25.00 ± 1.73 roots per explant, respectively. Their transformation rates were 55.61 ± 2.06%, 70.44 ± 2.61%, and 60.20 ± 4.35%, respectively. These differences may be related to gene-specific dosage effects, functional divergence among peanut homologs, differences in expression levels, or the fact that some regeneration regulators may function more effectively in shoot regeneration or somatic embryogenesis than in hairy-root induction. It is also possible that certain regulators require specific combinations with other factors to produce a stronger effect (Table 1).

**Figure 3:**
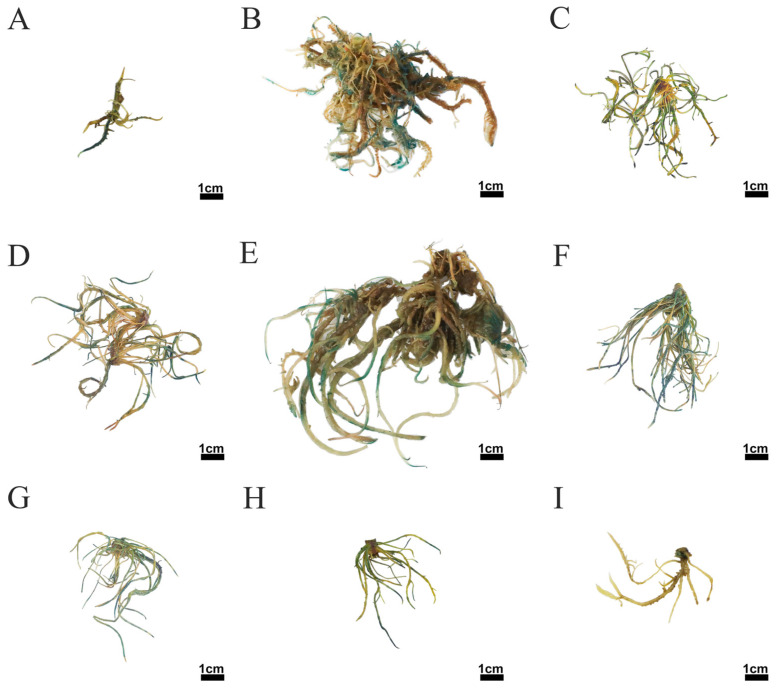
GUS staining images of hairy roots transformed by control and various carriers. (**A**): GUS staining of A (empty vector control) transgenic hairy roots. (**B**): GUS staining of B (*GRF-V829EQ* vector) transgenic hairy roots. (**C**): GUS staining of C (*GRF-2A7ZAY* vector) transgenic hairy roots. (**D**): GUS staining of D (*GRF-2A7ZAY-GIF-HK1F5C* vector) transgenic hairy roots. (**E**): GUS staining of E (*GRF-2A7ZAY_Mutant-GIF-HK1F5C* vector) transgenic hairy roots. (**F**): GUS staining of F (*GRF-FF6C67-GIF-HK1F5C* vector) transgenic hairy roots. (**G**): GUS staining of G (*WOX-PLVV0P* vector) transgenic hairy roots. (**H**): GUS staining of H (*WOX-ZS5XSZ-WOX-PLVV0P* vector) transgenic hairy roots. (**I**): GUS staining of non-transgenic hairy roots.

Differences among the tested constructs were evident in both the representative hairy-root phenotypes and the quantitative measurements of root number (Figure 3; Table 1 and Table S3). The empty-vector control produced relatively fewer hairy roots, with 18.67 ± 1.53 roots per explant at 4 weeks after infection. Construct C had a significantly higher number of roots than the control at 2 weeks; however, its transformation rate was lower. In the case of constructs B, G, and H, the differences in the number of roots were also significant, but the increase in transformation rate was not. In contrast, constructs D, E, and F produced more regenerated hairy roots, reaching 32.00 ± 1.00, 44.67 ± 1.53, and 31.33 ± 1.53 roots per explant, respectively (Table 1; Table S3). Among them, construct E showed the strongest effect, corresponding to a significant 2.4-fold increase in root number compared with the control. Additionally, this construct exhibited the highest GUS-positive transformation rate, showing a significant increase to 85.14 ± 2.94% compared with 64.58 ± 5.44% in the control (Table 1 and Table S3). Although root branching number and root thickness were not quantitatively measured in this study, visual inspection suggested that some developmental regulator treatments, especially constructs D, E, and F, produced more highly branched and apparently thicker hairy roots than the control (Figure 3). These results indicate that specific *GRF-GIF* combinations, particularly construct E, can enhance the number of regenerated hairy roots per explant and improve transformation efficiency in peanut.

## 3. Discussion

Hairy-root transformation mediated by *Agrobacterium rhizogenes* has been widely used in different plant species as a rapid system for root-specific gene-function analysis, metabolite production, and heterologous protein expression [26,27,28]. In these systems, plant explants are usually infected with activated *A. rhizogenes*, followed by co-cultivation and antibiotic selection to obtain sterile transgenic hairy roots. Because each transformed hairy root generally originates from an independent transformation event, this system can reduce the problem of chimerism compared with some whole-plant transformation approaches and provides useful material for rapid functional assays.

In peanut, however, genetic transformation remains relatively difficult because of genotype dependence, low transformation efficiency, and instability in regeneration [29]. Peanut is an allotetraploid legume derived from the wild diploid ancestors Arachis duranensis and Arachis ipaensis [30,31], and its complex genome may further contribute to the difficulty of establishing efficient transformation systems. Previous studies have established *A. rhizogenes*-mediated hairy-root transformation methods in peanut, showing that this approach is feasible for peanut gene-function studies [1,2,7]. In our preliminary work, we established a peanut hairy-root transformation system. Using 30-day-old rootless seedlings as explants, *Agrobacterium rhizogenes* K599 carrying a GUS reporter and developmental regulator constructs was used to infect peanut stem segments. Driven by the peanut endogenous promoter *AhUBQ4*, transgenic hairy roots were efficiently induced [32]. Developmental regulators such as *GRF*, *GIF*, and *WOX*, as well as their combinations, have been reported to improve transformation efficiency and shorten regeneration cycles in several plant species [14,33,34]. In our system, the empty-vector control produced 18.67 ± 1.53 roots per explant at 4 weeks after infection and showed a GUS-positive transformation rate of 64.58 ± 5.44%, indicating that the control vector already supported effective hairy-root induction and transformation. Compared with this baseline, several developmental regulator constructs further increased the number of regenerated roots per explant and/or the GUS-positive transformation rate, suggesting that these regulators can further improve the peanut hairy-root transformation platform.

Overexpression of developmental regulators has broad utility for improving plant regeneration and transformation. *GRF-GIF* fusion expression can substantially enhance transformation efficiency, increasing rates by 3.0–4.7-fold in wheat, rye, tomato, and citrus [14,35]. Overall, our results indicate that the tested developmental regulators had construct-dependent effects in peanut hairy-root transformation. In particular, construct E markedly enhanced both hairy-root production and GUS-positive transformation rate under our experimental conditions, whereas several other constructs showed only moderate or limited effects. These differences may be related to gene-specific dosage effects, functional divergence among peanut homologs, differences in expression levels, or the possibility that some regeneration regulators function more effectively in shoot regeneration or somatic embryogenesis than in hairy-root induction. It is also possible that certain regulators require specific combinations with other factors to produce a stronger effect [36]. Therefore, although the *GRF/GIF*-related and *WOX*-related constructs tested here provide useful candidates for further optimization, their effects should be interpreted in a construct-specific manner.

*WOX* genes also influence root development and differentiation: for example, *WOX11* overexpression in rice increases adventitious root formation, whereas *WOX11* mutants show root defects [37]. Plants expressing *TaWOX9* in *Arabidopsis* develop longer roots than controls, indicating a role in promoting root growth [38]. Consistent with these reports, our peanut *GRF*, *GRF-GIF* and *WOX* constructs improved hairy-root induction and growth, increasing roots per explant by 1.3–2.4-fold; furthermore, some vectors visually generated more branches and thicker roots. GUS staining confirmed stable transgene expression, supporting the use of these constructs as practical tools for peanut functional validation and metabolic engineering.

Hairy-root transformation provides a rapid and useful system for preliminary functional analysis in peanut, especially for testing CRISPR/Cas constructs, evaluating transgene activity, and studying root-related traits [9,32]. However, we also clarify its limitation: if transformed hairy roots cannot be regenerated into whole plants, this system cannot be used to directly evaluate traits involving shoots, reproductive development, seed formation, or whole-plant physiology. Therefore, an important practical goal is to develop a system in which transformed hairy roots not only show improved growth but can also regenerate into complete plants. In this context, our study provides an initial evaluation of several endogenous peanut developmental regulators and shows their potential effects on hairy-root induction and growth.

Although GRF-2A(396)-GIF-GUS produced transgenic hairy roots with high transformation efficiency and genetic stability, regenerating whole plants from hairy roots remains challenging. To date, stable transgenic plants regenerated from hairy roots have been reported for only a limited number of species, including sweet potato, radish, apple, and several medicinal plants [28,39,40]. Previous studies show that developmental regulators such as *WOX*, *GRF* and *GIF* play important roles in organ regeneration. For example, the *Wus2-ipt* combination can promote callus formation and directly induce regeneration buds at wound sites without exogenous hormones; using early-bolting genotypes, transgenic seeds can be obtained within 6–7 months, helping overcome radish transformation bottlenecks [34]. In sugar beet, *AtGRF5* promotes shoot formation and improves transformation of recalcitrant varieties, and *GRF5*/*GRF6*/*GRF9* can induce callus formation and differentiation in rapeseed [41]. Our results demonstrate that these developmental regulators can improve peanut hairy-root transformation and growth; future work should test their utility in peanut regeneration from hairy roots.

## 4. Materials and Methods

### 4.1. Plant Materials and Growth Conditions

**Figure 4:**
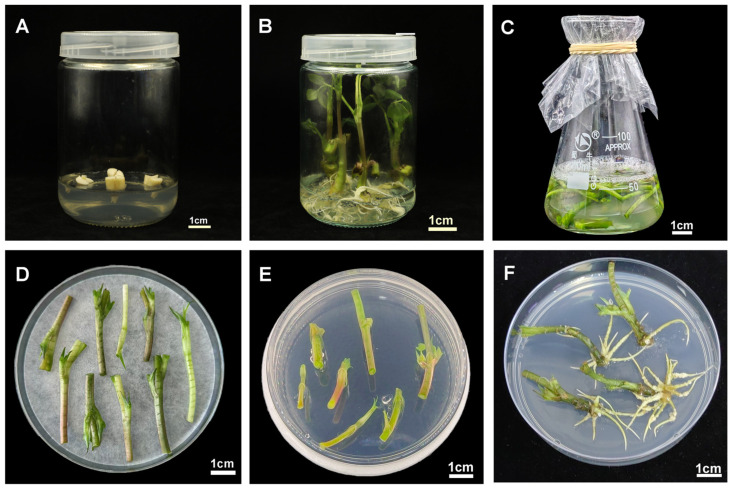
Transgenic *A. hypogaea* hairy roots obtained by *A. rhizogenes*-mediated transformation. (**A**): Seed germination. (**B**): Cultivate sterile seedlings for 30 days. (**C**): *A. rhizogenes* infection on explants. (**D**): Co-cultivation of explants after infection with *A. rhizogenes*. (**E**): Infused explants were transferred to induction culture medium. (**F**): The hairy roots produced.

The genetic transformation of hairy roots was studied by using the peanut variety Silihong preserved in Liu Xiaoqin’s laboratory of the Peking University Institute of Advanced Agricultural Sciences. We followed previously published protocols with modifications [2]. Seeds from four red-seeded peanut varieties were surface-rinsed and soaked overnight. After removing the seed coat under a laminar-flow hood, seeds were surface-sterilized in 75% ethanol for 1 min and then in 2.5% sodium hypochlorite (available chlorine) for 15 min. Seeds were rinsed five times with sterile water. Half of each cotyledon was removed with a scalpel, and cotyledons with intact embryos were placed on MS medium for germination. Seedlings were grown in a tissue-culture room under a 28 °C day/25 °C night cycle with 16 h light/8 h dark (Figure 4A). Thirty-day-old seedlings were used for infection experiments (Figure 4B). At this time, the sterile seedlings were vigorous and developed intact plants with roots, stems, leaves and other tissues. The roots and leaves of the tissue-culture seedlings were removed, leaving only the stems, and the stems were cut into 3–5 cm stem segments (1 or 2 axillary buds) and wounds were made with a scalpel (Figure 4C).

### 4.2. Identification and Cloning of GRF, GIF and WOX Genes in Peanut

Candidate members of the *GRF*, *GIF* and *WOX* gene families were identified from the *Arachis hypogaea* cv. Tifrunner genome (https://data.legumeinfo.org/Arachis/hypogaea/genomes/Tifrunner.gnm2.J5K5/, accessed on 10 November 2025) [42]. Protein sequences of reported GRF/GIF/WOX regulators from *Arabidopsis*, wheat, and rice were used as queries for homology-based searches against the peanut proteome. Putative candidates were further validated by confirming the presence of conserved domains using standard domain databases (NCBI CDD).

To infer evolutionary relationships and support candidate selection, representative GRF/GIF/WOX proteins from peanut and reference species were aligned and used to construct phylogenetic trees (Figure 1). Based on phylogenetic placement relative to known regulators (*AtGRF5*, *TaGRF4*, *OsGIF*, *AtWOX5*), several peanut candidates were prioritized for cloning and functional evaluation, including *GRF-V289EQ*, *GRF-2A7ZAY*, *GRF-FF6C67*, *GIF-HK1F5C*, *WOX-PLVV0P* and *WOX-ZS5XSZ* (Figure 1 and Figure 2).

The expression analysis of peanut *GRF* and *GIF* genes is shown (Figure S1). To examine the tissue-specific expression patterns of peanut *GRF* and *GIF* genes, publicly available transcriptome data were obtained from PeanutBase (https://www.peanutbase.org/). The expression profiles of candidate *GRF* and *GIF* genes were retrieved across different peanut tissues. Gene expression values were downloaded from the PeanutBase expression database and used to compare the relative expression levels of candidate genes in different tissues. The expression patterns, together with phylogenetic relationships, were used to guide the selection of representative *GRF* and *GIF* genes for vector construction and functional evaluation in the hairy-root transformation system.

Total RNA was extracted from young peanut leaves (Tifrunner) and reverse-transcribed into cDNA. Full-length coding sequences (CDSs) were amplified using gene-specific primers (Table S2), purified, cloned into an intermediate vector, and verified by Sanger sequencing prior to binary vector assembly.

### 4.3. Vector Construction

Binary vectors were constructed using a backbone reported previously [32]. As illustrated in Figure 2, all constructs were designed within the T-DNA region and included: (i) a hygromycin resistance cassette (Hyg) driven by the *CaMV35S* promoter for selection, and (ii) a GUS reporter cassette driven by the *AhUBQ4* promoter (*pAhUBQ4*) to facilitate rapid scoring of transformed hairy roots.

For functional testing of developmental regulators, individual CDSs and combinatorial modules were expressed under *pAhUBQ4* (Figure 2), including *GRF-V829EQ*, *GRF-2A7ZAY*, *WOX-PLVV0P*, and a dual-*WOX* construct (*WOX-ZS5XSZ-WOX-PLVV0P*) connected by a linker. To enable co-expression of *GRF* and *GIF*, *GRF-GIF* combinatorial constructs were generated by linking *GRF-2A7ZAY* (or *GRF-FF6C67*) with *GIF-HK1F5C* using a linker sequence (Figure 2). In addition, a miR396-related modified GRF module (as indicated by “Mutant miR396” in Figure 2) was generated to reduce miR396-mediated repression while maintaining the encoded protein sequence, and then assembled with *GIF-HK1F5C* for co-expression.

All plasmids were verified by restriction digestion and sequencing, and then introduced into *Agrobacterium rhizogenes* strain K599 using standard procedures. Transformed strains were selected on appropriate antibiotics before infection assays.

### 4.4. Hairy-Root Transformation

The prepared explants were used for *Agrobacterium rhizogenes* infection. For infection, *A. rhizogenes* was resuspended in infection medium (1/2 MS liquid medium supplemented with 100 μM acetosyringone) and adjusted to OD600 = 0.6; the suspension was incubated at 28 °C for 0.5–2 h. Explants were soaked in the infection suspension and shaken at 150 rpm for 30 min (Figure 4C). After infection, explants were washed 2–3 times with sterile water, blotted dry, and placed on 1/2 MS solid medium over filter paper for co-cultivation at 23 °C for 3 days (Figure 4D). Explants were then transferred to 1/2 MS solid medium containing timentin (300 mg/L) and hygromycin (20 mg/L) to suppress *Agrobacterium* and induce hairy roots (Figure 4E). Cultures were maintained at 28 °C under 16 h light/8 h dark, and the medium was refreshed every 2 weeks (Figure 4F). Explants from the same infection batch were transferred to MS medium and grown for 2 weeks before GUS staining. Each biological replicate included 10 explants, and each treatment was performed with three independent biological replicates.

### 4.5. GUS Histochemical Staining

Infected hairy roots were incubated in GUS staining solution (GUS staining kit, COOLABER, Beijing, China, SL7160) at 37 °C for 24 h. Samples were then rinsed 2–3 times with sterile water and photographed for documentation (Figure 4).

### 4.6. Data Statistics and Analysis

Data were recorded in Excel (Table S3) and analyzed using IBM SPSS STATISTICS 23.0 software. For each construct, the collected data included the number of regenerated hairy roots per explant at each observation time point, the number of explants producing hairy roots, the number of explants producing GUS-positive hairy roots, and the corresponding transformation rate. Each construct was evaluated using three independent biological replicates, and each replicate contained 10 explants, resulting in a total of 30 explants per treatment.

The number of regenerated roots per explant was counted by recording all independently emerged hairy roots from each infected stem segment. Clearly elongated roots were counted as regenerated hairy roots, whereas very short root primordia or callus-like protrusions without clear root elongation were not included.

The number of regenerated roots per explant was determined by counting independently emerged and clearly elongated hairy roots from each infected stem segment. For transformation assessment, a hairy root was considered GUS-positive when clear blue staining was observed in the regenerated root tissue, whereas blue staining restricted to the stem wound, residual explant tissue, or background precipitates was not counted. The transformation rate (%) shown in Table 1 was calculated as: (number of GUS-positive hairy roots/number of hairy roots) × 100%.

The data are presented as mean ± standard deviation (SD). Root regeneration traits and transformation rates were analyzed using one-way ANOVA followed by Duncan’s multiple range test. Differences were considered statistically significant at *p* < 0.05.

## 5. Conclusions

This study demonstrates that selected GRF-GIF developmental regulator constructs can improve *Agrobacterium rhizogenes*-mediated hairy-root transformation in peanut. Among the tested constructs, D (*GRF-2A7ZAY-GIF-HK1F5C* vector), E (*GRF-2A7ZAY_Mutant-GIF-HK1F5C* vector), and F (*GRF-2A7ZAY_Mutant-GIF-HK1F5C* vector) performed better than the empty-vector control, producing significantly higher GUS-positive transformation rates and a greater number of regenerated roots per explant. Construct E showed the strongest overall effect. Although root branch number and root thickness were not quantitatively measured, visual observation suggested that constructs D, E, and F produced more highly branched and apparently thicker hairy roots than the control. This improved hairy-root transformation protocol will be used in our future work for rapid functional validation of peanut candidate genes, testing of CRISPR/Cas genome-editing constructs, and further optimization of regeneration strategies from transformed hairy roots.

## Figures and Tables

**Table 1:** Root regeneration and transformation rates recorded after injection.

Constructs *	Number of Regenerated Roots per Explant After One Week	Number of Regenerated Roots per Explant After 2 Weeks	Number of Regenerated Roots per Explant After 3 Weeks	Number of Regenerated Roots per Explant After 4 Weeks	Transformation Rate %
A	0.00 ± 0.00 d **	2.67 ± 0.58 e	11.67 ± 0.58 g	18.67 ± 1.53 e	64.58 ± 5.44 cd
B	1.67 ± 0.58 c	6.33 ± 0.58 d	21.00 ± 1.00 d	34.00 ± 1.00 b	67.69 ± 2.00 c
C	0.67 ± 0.58 cd	6.67 ± 0.58 d	17.33 ± 0.58 e	27.00 ± 1.00 d	55.61 ± 2.06 f
D	3.67 ± 0.58 b	8.67 ± 0.58 c	23.67 ± 1.53 c	32.00 ± 1.00 bc	78.18 ± 2.45 b
E	6.33 ± 1.53 a	16.33 ± 1.53 a	36.33 ± 0.58 a	44.67 ± 1.53 a	85.14 ± 2.94 a
F	3.00 ± 1.00 b	10.33 ± 0.58 b	25.67 ± 0.58 b	31.33 ± 1.53 c	76.72 ± 3.69 b
G	1.33 ± 0.58 cd	5.67 ± 0.58 d	15.00 ± 1.00 f	27.00 ± 1.00 d	70.44 ± 2.61 c
H	1.00 ± 0.00 cd	6.33 ± 1.53 d	17.67 ± 1.15 e	25.00 ± 1.73 d	60.20 ± 4.35 de

* (A): empty vector control; (B): *GRF-V829EQ* vector; (C): *GRF-2A7ZAY* vector; (D): *GRF-2A7ZAY-GIF-HK1F5C* vector; (E): *GRF-2A7ZAY_Mutant -GIF-HK1F5C* vector; (F): *GRF-FF6C67-GIF-HK1F5C* vector; (G): *WOX-PLVV0P* vector; (H): *WOX-ZS5XSZ-WOX-PLVV0P* vector. ** Different lowercase letters in the same column indicated the significant difference at *p* ≤ 0.05.

## Data Availability

All data generated or analyzed during this study are included in this published article and its Supplementary Information Files.

## Supplementary Materials

The following supporting information can be downloaded at: https://www.mdpi.com/article/10.3390/plants15121889/s1, Table S1. summarizing all constructs, including the full name, included genes, promoter, use of 2A peptide or linker, presence of the miR396-resistant modification, and reporter gene for each construct. Table S2. Primers and T-DNA sequences used in this study. Table S3. Hairy-root-related data. Figure S1. Expression levels of *GRF* and *GIF* in different tissues.

## Abbreviations

The following abbreviations are used in this manuscript:

*GRF*	Growth-Regulating Factor
*GIF*	GRF-Interacting Factor
*WOX*	WUSCHEL-related homeobox
