# Supplementary figures and images for: Enhancing Agrobacterium-Mediated Hairy-Root Transformation Efficiency in Peanut Through the Application of GRF, GIF and WOX Genes
Source: Plants (Basel). 2026 Jun 18;15(12):1889. doi: 10.3390/plants15121889 (PMC13306245; doi:10.3390/plants15121889)

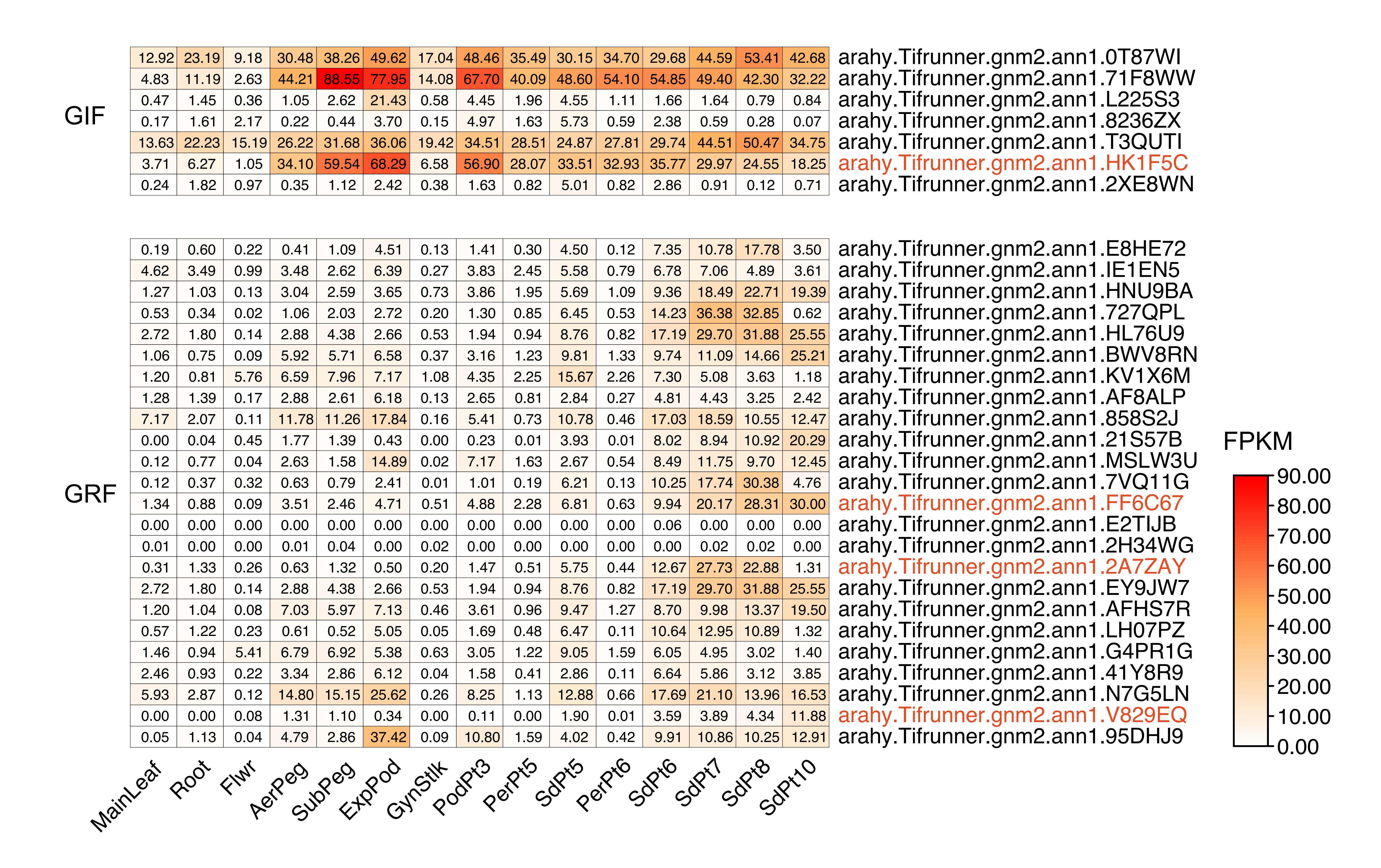

Supplement: Supplementary file 1 [file plants-15-01889-s001.zip › Figure S1.jpg]
